# Between-course targeting of methotrexate exposure using pharmacokinetically guided dosage adjustments

**DOI:** 10.1007/s00280-013-2206-x

**Published:** 2013-06-13

**Authors:** Jennifer L. Pauley, John C. Panetta, Kristine R. Crews, Deqing Pei, Cheng Cheng, John McCormick, Scott C. Howard, John T. Sandlund, Sima Jeha, Raul Ribeiro, Jeffrey Rubnitz, Ching-Hon Pui, William E. Evans, Mary V. Relling

**Affiliations:** 1 Department of Pharmaceutical Sciences, St. Jude Children’s Research Hospital, 262 Danny Thomas Place, Memphis, TN 38105-3678 USA; 2 Department of Biostatistics, St. Jude Children’s Research Hospital, 262 Danny Thomas Place, Memphis, TN 38105-3678 USA; 3 Department of Oncology, St. Jude Children’s Research Hospital, 262 Danny Thomas Place, Memphis, TN 38105-3678 USA; 4 Colleges of Medicine and Pharmacy, University of Tennessee, Memphis, TN USA

**Keywords:** Methotrexate, Acute lymphoblastic leukemia, Pharmacokinetics, Individualized therapy

## Abstract

**Purpose:**

It is advantageous to individualize high-dose methotrexate (HDMTX) to maintain adequate exposure while minimizing toxicities. Previously, we accomplished this through within-course dose adjustments.

**Methods:**

In this study, we evaluated a strategy to individualize HDMTX based on clearance of each individual’s previous course of HDMTX in 485 patients with newly diagnosed acute lymphoblastic leukemia. Doses were individualized to achieve a steady-state plasma concentration (Cpss) of 33 or 65 μM (approximately 2.5 or 5 g/m^2^/day) for low- and standard-/high-risk patients, respectively.

**Results:**

Individualized doses resulted in 70 and 63 % of courses being within 20 % of the targeted Cpss in the low- and standard-/high-risk arms, respectively, compared to 60 % (*p* < 0.001) and 61 % (*p* = 0.43) with conventionally dosed therapy. Only 1.3 % of the individualized courses in the standard-/high-risk arm had a Cpss greater than 50 % above the target compared to 7.3 % (*p* < 0.001) in conventionally dosed therapy. We observed a low rate (8.5 % of courses) of grade 3–4 toxicities. The odds of gastrointestinal toxicity were related to methotrexate plasma concentrations in both the low (*p* = 0.021)- and standard-/high-risk groups (*p* = 0.003).

**Conclusions:**

Individualizing HDMTX based on the clearance from the prior course resulted in fewer extreme Cpss values and less delayed excretion compared to conventional dosing.

**Electronic supplementary material:**

The online version of this article (doi:10.1007/s00280-013-2206-x) contains supplementary material, which is available to authorized users.

## Introduction

Improvements in cure rates for childhood acute lymphoblastic leukemia (ALL) are due partly to the use of risk-directed chemotherapy [1–5]. One important element of chemotherapy is high-dose methotrexate (HDMTX) (doses ≥1 g/m^2^) with leucovorin rescue [3, 6–9]. High doses of methotrexate (5 g/m^2^) improve the outcome of patients with T-lineage ALL, consistent with the fact that T-lineage blasts accumulate less methotrexate polyglutamates than blasts of B-lineage ALL, thereby requiring higher serum concentrations to achieve the same cytotoxic effect [6, 10–12]. In addition, methotrexate dosages higher than 1 g/m^2^ are beneficial for patients with B-lineage ALL [13–16]. However, HDMTX has been associated with potentially severe toxicities, [17–20] although the introduction of pretreatment prehydration, urinary alkalinization, routine monitoring of serum methotrexate concentrations, and the incorporation of leucovorin rescue has decreased their incidence [21–25]. High plasma methotrexate concentrations are also associated with increased toxicity which may delay subsequent courses of chemotherapy [22, 26, 27]. Therefore, it is desirable to maintain plasma concentrations within the putative cytotoxic range for leukemic blasts [28] and below those associated with significant toxicity. We previously conducted a prospective randomized trial in children with ALL which demonstrated that when HDMTX doses were adjusted during the 24-h drug infusion to achieve desired plasma exposure levels, relapse rates were lower compared to conventional fixed doses of methotrexate based on body surface area [13]. Another study in patients with relapsed ALL showed that individualizing doses decreased inter-patient variability and avoided potentially toxic methotrexate concentrations [29].

However, adjusting doses of HDMTX during an infusion requires extremely fast turn-around time for analysis of plasma methotrexate concentrations, estimating pharmacokinetic parameters, and implementing adjusted doses. Therefore, the objectives of this follow-up study were to evaluate the feasibility of an approach that individualized HDMTX dosage based on the pharmacokinetics of each individual patient’s previous course of methotrexate, with 14 or more days between each course and to assess the acute toxicities associated with HDMTX.

## Patients and methods

Between June 2000 and October 2007, 501 patients were enrolled on St. Jude Total Therapy Study XV for ALL [1]. Three patients were subsequently excluded based on a revised diagnosis of myeloid leukemia, and two patients did not receive HDMTX during consolidation therapy. In addition, for this analysis, 12 patients with Down syndrome were excluded because they received lower doses of methotrexate (500 mg/m^2^) that were not individualized based on pharmacokinetics. During this front-line study of childhood ALL, patients were randomly assigned to receive initial treatment (window) with HDMTX (1 g/m^2^) over a period of 4 or 24 h [30]. Four days later, remission-induction therapy was begun with prednisone, vincristine, daunorubicin, asparaginase, cyclophosphamide, mercaptopurine and cytarabine. Risk classification was based on presenting characteristics and treatment response to remission-induction therapy, and patients were assigned to the low-, standard- or high-risk categories [1]. Central nervous system-directed therapy with triple intrathecal therapy was given based on the patient’s central nervous system status [1].

The 6-week induction period was followed by consolidation, consisting of four courses of HDMTX given every other week together with triple intrathecal therapy with methotrexate, hydrocortisone and cytarabine on the day of HDMTX and daily oral mercaptopurine at 50 mg/m^2^/day at bedtime for the 8 weeks of consolidation. Serum chemistries were required to be within normal limits prior to receipt of HDMTX. Methotrexate doses were individualized using the pharmacokinetic parameters estimated from the individual’s previous course of HDMTX. If the patient had not received HDMTX as part of window therapy, then their first course of HDMTX in consolidation was a fixed dose of 2.5 mg/m^2^ (low-risk arm) or 5 mg/m^2^ (standard-/high-risk arm) infused over 24 h. Patients on the low-risk treatment arm had doses individualized to achieve a steady-state plasma concentration (Cpss) of 33 μM (the average Cpss expected for patients receiving 2.5 g/m^2^/24 h based on extensive prior pharmacokinetic estimates in children with ALL) [13]. Those on the standard-/high-risk arm were individualized to a Cpss of 65 μM (the average Cpss expected for patients receiving 5 g/m^2^/24 h, based on prior pharmacokinetic estimates) [13]. Patients received prehydration containing sodium bicarbonate starting the evening prior (at 100 or 125 mL/m^2^/h for low risk or standard/high risk, respectively). In some cases, prehydration was administered for a minimum of 2 h prior at 200 mL/m^2^/h with a sodium bicarbonate bolus. This occurred in extenuating circumstances such as when patients were not able to arrive in our clinic the evening prior to treatment. The HDMTX was not started until the urine pH was ≥6.5. Intravenous fluids continued until at least 42 h after the start of methotrexate. HDMTX was given as a 10 % loading dose over 1 h, with the remaining 90 % administered over 23 h. Urine pH was monitored with each void, and an IV sodium bicarbonate bolus was given if the urine pH was ≤6 [22].

All patients had methotrexate concentrations measured by fluorescence polarization immunoassay (TDx/TDxFLx Systems, Abbot Laboratories, Abbot Park, IL, USA) prior to the dose and at 6, 23 and 42 h from the infusion start. The lower limit of quantification of the assay was 0.03 μM.

### Leucovorin dosing

Leucovorin rescue was started at 42 h from the beginning of the HDMTX infusion (Supplemental Table 1). Those on the low-risk arm received 10 mg/m^2^ of leucovorin every 6 h for 5 doses and those on the standard-/high-risk arm received 15 mg/m^2^ of leucovorin every 6 h for 5 doses. Leucovorin doses were increased for patients with delayed excretion of methotrexate, defined as methotrexate concentrations >1 μM at 42 h (Supplemental Table 1). For those with delayed excretion, plasma methotrexate concentrations were monitored, and leucovorin was continued until plasma methotrexate concentrations were <0.1 μM. Patients with changes to their clinical status (e.g., an increase in serum creatinine; early mucositis; evidence of pleural effusions or ascites; significantly delayed methotrexate excretion) were followed until they achieved an undetectable plasma methotrexate concentration (<0.03 μM).

### Pharmacokinetic modeling

Methotrexate pharmacokinetic parameters were estimated by fitting a two-compartment model [13, 31–33] to each individual’s data set using the a posteriori probability estimation method implemented in ADAPT II [34]. Extensive prior studies established the prior distribution of the methotrexate pharmacokinetic parameters and allowed the implementation of our Bayesian approach using relatively sparse sampling (3–4 samples per course) in this and our prior studies [13]. The prior pharmacokinetic parameters (mean ± SD) were as follows: *k*_e_ (0.70 ± 0.22 1/h); *V* (9.03 ± 4.70 L/m^2^); *k*_cp_ (0.080 ± 0.050 1/h); and *k*_pc_ (0.11 ± 0.0038 1/h). Clearance (CL) was calculated as *k*_e_·*V*. Note that volume and clearance were always normalized for body surface area.

### Methotrexate dose individualization

The individualized methotrexate dose for each course was determined as follows (Supplemental Figure 1):

$$ {\text{Targeted}}\,{\text{dose }}\left( {{\text{mg}}/{\text{m}}^{ 2} } \right) = {\text{Infusion}}\,{\text{Length }}\left( {\text{h}} \right)\cdot{\text{Predicted}}\,{\text{CL }}\left( {{\text{L}}/{\text{h}}/{\text{m}}^{ 2} } \right)\cdot{\text{Cpss }}\left( {\mu {\text{M}}} \right)/\left( { 2. 2\cdot\left( { 1- {\text{fraction loading dose}}} \right)} \right) $$

where the infusion length was 23 h (24–1 h loading dose infusion), the fraction loading dose was 0.1 (or 10 %), and the predicted clearance was defined as follows. For the low-risk arm, the predicted clearance was assumed to be equal to the clearance of the previous course of methotrexate. For the first 53 patients in the standard-/high-risk arms, the predicted clearance was also assumed to be equal to the clearance of the previous course of HDMTX. However, due to the lower success of individualization by this approach for patients on the standard-/high-risk arm (see “Results”), we investigated whether serum chemistries (obtained within 24 h before the targeted course) or patient demographics could help improve our ability to predict the MTX clearance in the patients on the standard-/high-risk arm and thus more accurately target individuals. We considered serum chemistries as possible predictors of MTX clearance because when MTX is infused over 24 h, both renal and hepatic function play a role in clearance. Approximately 40 % of HDMTX is cleared non-renally, mostly via hepatic metabolism to 7-hydroxymethotrexate [35], and both SGPT [36] and serum bilirubin [22] have been associated with MTX clearance. Therefore, using the data from the initial group of standard-/high-risk patients on the protocol, we built a linear model with the previous course MTX clearance, serum chemistries and demographics as potential predictors of the current course MTX clearance, using stepwise linear regression (forward selection followed by backward elimination) [37]. We found a significant association between methotrexate clearance and serum concentrations of creatinine, bilirubin and SGPT (Supplemental Figure 2), and we estimated the predicted clearance for patients in the standard-/high-risk arms based on a linear function of the clearance of the previous course of methotrexate along with current serum concentrations of creatinine, bilirubin and SGPT (Supplemental Figure 1). If the clearance for the previous course of methotrexate was >125 mL/min/m^2^, we used only the previous clearance as the predicted clearance because it was a more accurate predictor than the combination of prior clearance and serum creatinine, bilirubin and SGPT concentrations in such cases (Supplemental Figure 1). Among patients on the low-risk arm, simulations did not demonstrate an improvement in the prediction of clearance if serum chemistries were incorporated.

We also simulated the methotrexate exposure (Cpss and 42 h methotrexate concentration) that would have been achieved had patients received the conventional dose (i.e., 2.5 g/m^2^ for low risk and 5 g/m^2^ for standard/high risk), using the estimated methotrexate pharmacokinetic parameters for each course in each individual.

### Targeting success

We measured targeting success by comparing the proportion of courses that were successfully targeted (defined by ±20 % of the target Cpss [33 μM for low risk and 65 μM for standard/high risk]) by pharmacokinetically based doses vs fixed doses (simulated at 2.5 or 5.0 g/m^2^ for low or standard/high risk, respectively) using the McNemar’s *Χ* ^2^ test. This ±20 % window was based on our prior intra-course targeting study [13]. In addition, all targeted patients had their doses adjusted to attempt to achieve the target concentration, even if their predicted Cpss was within 20 % of the target Cpss.

### Toxicities

Toxicities during HDMTX consolidation were graded using the NCI Common Toxicity Criteria version 2.0 (http://ctep.cancer.gov/protocolDevelopment/electronic_applications/ctc.htm); we recorded any grade 3–4 toxicities in the following categories: allergy/immunology, cardiovascular, constitutional symptoms, dermatology/skin, gastrointestinal, hepatic, infection/febrile neutropenia, metabolic/laboratory, musculoskeletal, neurology, pain, pulmonary, renal/genitourinary. We determined whether methotrexate exposure (Cpss or 42 h concentration), delayed methotrexate excretion (42 h methotrexate concentration >1 μM), leucovorin dose, or success of targeting methotrexate was associated with the incidence of grade 3 or greater toxicity using a univariate generalized estimating equation (GEE) model.

## Results

### Low-risk arm individualization

**Fig. 1:**
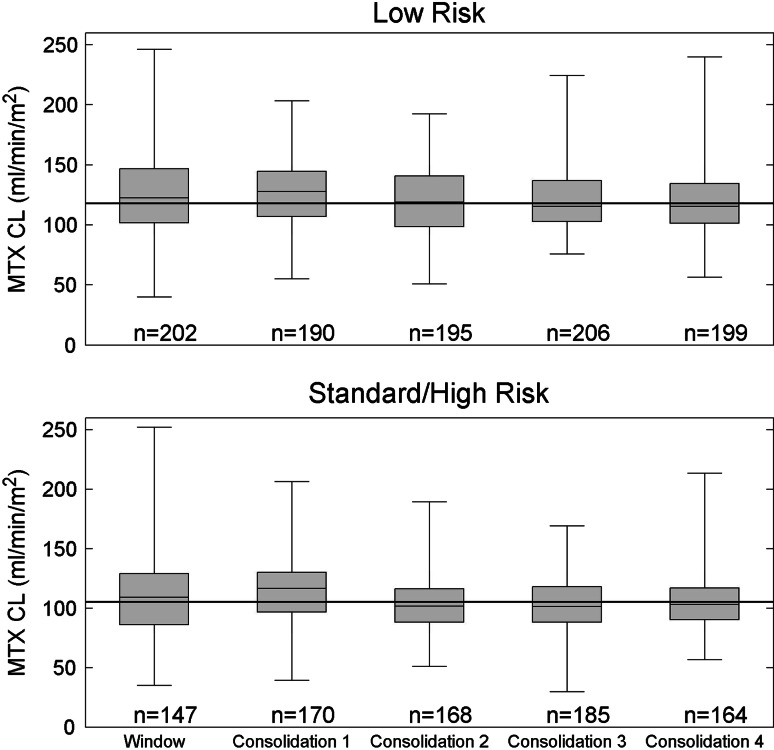
Methotrexate clearance, MTX CL (ml/min/m^2^), by course and risk arm. The *horizontal line* in each *box* represents the median, the *shaded boxes* represent the quartiles, and the whiskers represent the range observed in patients for each course. The *solid horizontal line* across all courses represents the population clearance for all courses

Of the 915 consolidation HDMTX courses delivered to the 233 patients on the low-risk arm, 754 courses in 220 patients were individualized based on the methotrexate clearance from the previous course. The patient demographics are described in Supplemental Table 2. The remaining doses were not pharmacokinetically based due to various reasons, including no previous clearance data being available, doses being lowered due to prior toxicities, or other clinical issues such as unstable renal function. The median dose administered for these pharmacokinetically based courses was 2.8 g/m^2^, with a range of 0.9–5.3 g/m^2^, and the median Cpss for the pharmacokinetically based courses was 33 μM with a range from 15.6 to 92.3 μM. This median Cpss observed with pharmacokinetically based doses was 10 % higher than the simulated median Cpss for patients on this study given a fixed dose of 2.5 g/m^2^ (*p* < 0.001). Figure 1 shows the methotrexate clearance subdivided by course. The overall population clearance, inter-individual (IIV) and inter-occasion (IOV) variability of the clearance were 117.9 ml/min/m^2^, 17.7 and 15.7 %, respectively.

**Fig. 2:**
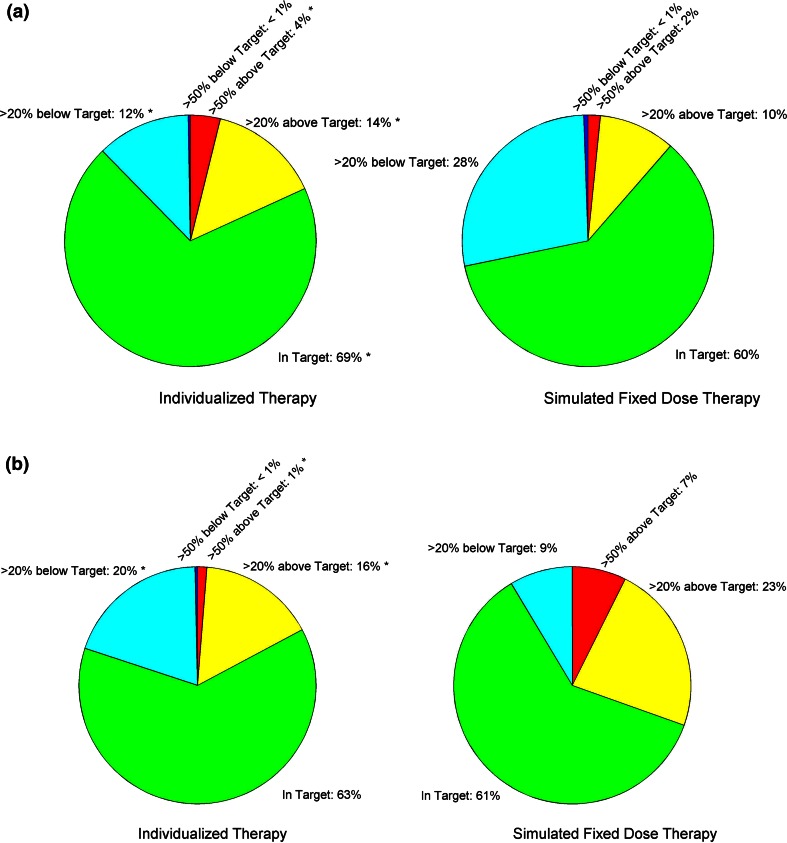
Percentage of courses based on achieved MTX plasma steady-state concentrations (Cpss) (individualized therapy) compared to the percentage predicted based on conventional dosing (simulated for fixed doses). The groups are defined as follows: *Dark Blue* Cpss greater than 50 % below target Cpss, *Light Blue* Cpss between 20 and 50 % below target, *Green* Cpss within ±20 % of the target, *Yellow* Cpss between 20 and 50 % above target, *Red* Cpss greater than 50 % above target. *Significance (*p* < 0.001) in the difference in the proportion of courses between individualized therapy relative to simulated fixed dose therapy (otherwise *p* > 0.1). **a** Low-risk arm: target concentration: 33 μM. **b** Standard-/high-risk arm: target concentration: 65 μM

For the low-risk arm, a higher proportion (69.5 %) of pharmacokinetically based courses were within target (defined as ±20 % of the Cpss of 33 μM) compared to that estimated (60.3 %) had patients received a fixed dose of 2.5 g/m^2^ (*p* < 2 × 10^−4^; Fig. 2).

There was substantial IIV and IOV in methotrexate clearance for both window and consolidation therapy. When subdivided by course, the IOV during the window and first two consolidation courses was higher than that for the later consolidation courses (15.8 vs. 11.3 %; *p* < 0.03). When comparing the change in clearance ([previous clearance-current clearance]/current clearance) for pharmacokinetically based courses 1 and 2 vs courses 3 and 4, we observed more variability in the change in clearance for the first two courses compared to the last two courses ([−23, 34 %] and [−20, 28 %] (10th, 90th percentile), respectively; *p* < 0.03). These differences in IOV over time made it more difficult to accurately target courses 1 and 2 than the latter two consolidation courses. We observed a higher proportion of courses with Cpss within the target range for individualized doses in the latter two consolidation courses: for consolidation 3 and 4: 9.6, 74.6, and 15.8 % of courses were below target, within target and above target, respectively; vs for consolidation 1 and 2: 15.5, 63.6, and 20.9 % of courses were below, within and above target, respectively (*p* < 0.001).

To assess whether individualizing doses minimized the proportion of courses with extremes of plasma concentrations of methotrexate, we compared the percent of courses that yielded Cpss that were outside ±50 % of the target (i.e., Cpss <16.5 μM or >49.5 μM for patients on the low-risk arm, Fig. 2) for individualized courses vs that simulated with conventional fixed dosing. Due to the higher than expected clearance in patients in this arm, our simulations show that the 2.5 g/m^2^ fixed dose actually would have resulted in fewer courses with Cpss above 49.5 μM compared to individualized doses (1.6 vs. 3.8 %; *p* < 0.005), while there was no difference in courses with Cpss below 16.5 μM (0.5 vs. 0.3 %; *p* > 0.1).

Finally, we observed a lower proportion of pharmacokinetically based courses with delayed excretion compared to simulated fixed dose courses (7.4 vs. 8.9 %, respectively, *p* < 0.04).

### Standard-/high-risk arm individualization

Of the 965 courses of consolidation HDMTX delivered to 252 patients on the standard-/high-risk arm, 97 courses in 53 patients had doses individualized based on the clearance from the previous course; subsequently, for 627 courses in 224 patients, doses were individualized based on the methotrexate clearance from the previous course along with current serum creatinine, bilirubin, and SGPT. The patient demographics are described in Supplemental Table 2. The remaining courses were not pharmacokinetically based due to various reasons including no previous clearance being available, doses being lowered due to toxicities, or other clinical issues, such as unstable renal function. The median dose for courses individualized using only the previous clearance was 4.5 g/m^2^ (range 1.5–6.7 g/m^2^), and for courses individualized using both the previous clearance and serum chemistries was 4.6 g/m^2^ (range 2.9–8.6 g/m^2^). The median [10th–90th percentile] Cpss for the courses targeted using only the previous clearance was 63 μM [34, 84 μM] while that for the courses individualized, using both the previous clearance and serum chemistries was 63 μM [46, 83 μM]. This median Cpss observed with pharmacokinetically based doses was 10 % lower than the simulated median Cpss for patients on this study given a fixed dose of 5 g/m^2^ (*p* < 0.001). Figure 1 shows the methotrexate clearance subdivided by course. The overall population clearance, IIV, and IOV variability of the clearance were 105.2 ml/min/m^2^, 17.5, and 16.9 %, respectively.

As in the low-risk arm, the IOV for methotrexate clearance for the window and first two consolidation courses was higher than that for the later consolidation courses (18.2 vs. 10.7 %; *p* < 0.002). Again, the change in clearance for individualized courses 1 and 2 displayed more variability than for courses 3 and 4 ([−29, 35 %] and [−21, 24 %] (10th, 90th percentile), respectively; *p* < 0.003). As with the low-risk arm, the high IOV in the clearance for early courses made it more difficult to accurately individualize the first two consolidation courses.

For the first 97 courses given to patients on the standard-/high-risk arm, when methotrexate was dosed based only on the prior clearance, there was a trend for a lower proportion of courses to achieve Cpss in the target range compared to a conventional dose of 5 g/m^2^ (42.3 vs. 55.7 %; *p* = 0.087) and also compared to the subsequent 627 courses, when methotrexate was dosed based on the previous clearance plus the current serum chemistries (42.3 vs. 62.8 %, *p* < 0.001). Thus, our final approach was to base doses for the standard-/high-risk group on both the previous clearance and the current serum chemistries. In the final analysis, there was not a significant difference in the proportion of pharmacokinetically based courses with Cpss that were within target (*n* = 627) compared to that of conventional dosing of 5 g/m^2^ (62.8 vs. 60.9 %, respectively; *p* = 0.43; Fig. 2). However, there was a significantly lower proportion of courses with Cpss above the targeted range with individualized dosing compared to that predicted with conventional dosing (17.2 vs. 30.5 %, respectively; *p* < 0.001; Fig. 2). Again, due to the larger IOV in methotrexate PK for the early compared to later courses, we observed a trend toward (*p* = 0.12) better success in achieving target Cpss with individualized doses in the latter two consolidation courses (consolidation 3 and 4: 17.8, 65.0, and 17.2 % below, within and above target, respectively) compared to consolidation 1 and 2 (22.7, 60.1, and 17.3 % below, within and above target, respectively).

Next, to assess how well the individualization avoided extreme plasma concentrations of MTX, we compared how many courses yielded Cpss outside ±50 % of the target (i.e., Cpss <32.5 or >97.5 μM). By using pharmacokinetically based doses, the proportion of courses with extremely high Cpss was lower than that simulated with a fixed dose (1.3 vs. 7.3 %; *p* < 10^−8^, Fig. 2), while there was no difference in the frequency of courses with extremely low Cpss (0.3 vs 0.0 %; *p* = 0.48, Fig. 2).

Finally, we observed a lower proportion of courses with delayed excretion when individualized dosing was used compared to what we would have observed had we given fixed doses (14.7 vs. 19.8 %, respectively, *p* < 0.001).

### Toxicity

The overall rate of any grade 3–4 toxicity in this study was low (160 events from 1,880 methotrexate consolidation courses or 8.5 % of courses) (Table 1). Infection/febrile neutropenia and gastrointestinal toxicities were the most common events, occurring in 5.2 and 3.0 % of courses, respectively. As expected based on the protocol guidelines (Supplemental Table 1), there was a 4.3-fold increase in the total leucovorin dose in patients with delayed excretion for the low-risk arm (*p* < 0.001) and a 2.2-fold increase for the standard- and high-risk arms (*p* < 0.001).

**Table 1:** Grade 3 or 4 toxicities during consolidation therapy

	LR individualized	LR not individualized	S/HR individualized	S/HR not individualized
Methotrexate courses (*n*)	754	161	627	338
Patients (*n*)	220	101	224	176
Courses with infection/febrile neutropenia (*n*)	33	10	29	26
Course with gastrointestinal toxicities (*n*)	13	3	19	22
Courses with any grade 3 or 4 toxicity [*n* (%)]	52 (6.9)	14 (8.7)	50 (8.0)	44 (13.0)

*LR* Low-risk therapy arm, *S/HR* standard-/high-risk therapy arm, *Individualized* courses for which the methotrexate dose was adjusted based on the prior course’s pharmacokinetic parameters, *Not Individualized* courses for which the methotrexate was dosed conventionally based on body size

We investigated the relationship between methotrexate Cpss, methotrexate 42-h concentration, targeting success (Cpss ±20 % of target), leucovorin dose, methotrexate delayed excretion, and risk of gastrointestinal toxicity. In the low-risk arm, higher methotrexate Cpss corresponded to a higher odds (5 % higher for every 1 μM increase in the Cpss) of having a grade 3 or greater gastrointestinal toxicity (OR 1.05; 95 % CI 1.01–1.09, *p* = 0.021) (Supplemental Table 3). In the standard-/high-risk arm, methotrexate delayed excretion was associated with gastrointestinal toxicities (OR 3.02; 95 % CI 1.45–6.32, *p* = 0.003). No other measures of methotrexate exposure or targeting success related significantly to toxicity (*p* > 0.05 in all cases).

Of a total of 1,880 consolidation courses in 484 patients receiving HDMTX, severe delayed excretion (requiring glucarpidase) occurred for a single course in each of only 4 patients (0.8 % of patients and 0.21 % of courses). All 4 of these patients received targeted dosing on the standard-/high-risk arm and had a large (24–71 %) decrease in their MTX clearance between their previous course of MTX (used for targeting) and the course with severe delayed excretion. Patients requiring glucarpidase were eligible to be targeted with future courses. MTX concentrations used to target were obtained prior to administration of glucarpidase; therefore, falsely elevated concentrations due to the glucarpidase were not an issue.

## Discussion

HDMTX is an important chemotherapeutic agent that contributes to the high cure rate of pediatric ALL [6, 7, 38]. Higher plasma methotrexate concentrations following HDMTX have been associated with a lower risk of relapse [10, 11]; however, high concentrations may lead to delayed excretion, increased toxicity and delays in receiving subsequent courses of chemotherapy [22]. In a previous study, we have shown that adjusting the dose of methotrexate during the 24-h infusion, to account for inter-individual differences in drug clearance and to achieve a target Cpss, improved the outcome in children with B-lineage leukemia [10, 13, 29]. Other studies have retrospectively investigated therapeutic drug-monitoring approaches to predict MTX concentrations after a dose to help determine appropriate leucovorin rescue [31]. In the current study, we tested whether we could successfully individualize doses of HDMTX based on clearance estimates from the prior course of MTX for each child; this method relies on the assumption that IOV is low enough that prior clearance accurately reflects current clearance. However, we observed high IOV, particularly for the first and second course of HDMTX. Recognizing this large IOV, we modified our targeting strategy in the standard-/high-risk arm to include not only prior pharmacokinetic parameters, but also serum chemistries (drawn within 24 h of each planned HDMTX course) as a surrogate for changing methotrexate clearance. Simulations showed that serum chemistries would not have improved targeting in the low-risk arm.

Targeting doses based on the prior course’s pharmacokinetic parameters was moderately successful. For the low-risk arm, more courses had Cpss that were within target (70 %) compared to simulated conventional dosing (60 %; *p* < 0.001). The median targeted dose was 11 % higher than the conventional 2.5 g/m^2^ dose without an increase in delayed excretion. For the standard-/high-risk arm, even after incorporation of current serum chemistries, we were not successful in improving the percentage of courses with Cpss in the target range (±20 %) over that achieved with conventional dosing. We attribute this finding to the higher IOV in clearance relative to the low-risk arm, which could be exacerbated in patients on the standard-/high-risk arm because clearance of higher doses of MTX is more susceptible to transient insults (e.g., changes in hydration, drug interactions). Nonetheless, individualized dosing decreased the frequency of both extreme Cpss and of delayed excretion in patients on the standard-/high-risk arm. For courses given to patients on the standard-/high-risk arm, only 1 % of those that received individualized dosing had Cpss >50 % above target compared to 7 % of courses with simulated conventional dosing (*p* < 0.001). This translated into a lower proportion of individualized courses with delayed excretion.

Gastrointestinal toxicities are known side effects of HDMTX. In our prior study comparing conventional versus individualized chemotherapy, with a Cpss of only 20 μM, there was no difference in toxicity between the two groups [13]. In the current study, using much higher target concentrations of 33 and 65 μM, minimal toxicity was noted following HDMTX. Overall, 5.2 % of courses were followed by grade 3–4 infection/febrile neutropenia and 3.0 % was followed by grade 3–4 gastrointestinal toxicities. These toxicity rates were low compared to other studies. In the POG9404 study, patients that received HDMTX (5 g/m^2^ over 24 h) had a 17.8 % incidence of mucositis and 66.2 % incidence of infection [7]. For patients on the LAL-SHOP 99 and 2005 protocols receiving HDMTX of 3 or 5 g/m^2^, there was an 11 % incidence of mucositis [39]. D’Angelo and colleagues [40] reviewed patients enrolled on AIEOP-ALL studies 91, 95 and 00 and reported that 68.2 % of patients developed grade 3–4 hematologic and non-hematologic toxicity combined after HDMTX doses of 2 or 5 g/m^2^. The BFM study NHL-BFM95 reported incidences of 36 % for grade 3 and 43 % for grade 4 mucositis in patients with B-cell neoplasms receiving 5 g/m^2^ over 24 h; perhaps this high frequency is partly attributed to only three doses of leucovorin being administered after HDMTX compared to our 5 doses [41]. Leucovorin dose was not a risk factor for toxicity in either our LR or S/HR arms, despite being significantly higher in those with elevated 42-h concentrations. Thus, our data suggest that the algorithms we used (Supplemental Table 1) for adjusting leucovorin doses based on plasma methotrexate concentrations, combined with our targeting strategy, were effective in producing a low frequency of adverse effects. It should also be noted that the relapse rate on the Total XV protocol was very low [1].

Renal excretion plays a major role in methotrexate elimination, and methotrexate itself can cause acute nephrotoxicity. Close monitoring of patients receiving methotrexate is imperative. At St. Jude, close monitoring of fluid status, urine output, urine pH, laboratory values, methotrexate concentrations [22, 23, 42] and drug interactions (through review of the patient’s electronic record) in order to prevent delayed excretion and toxicity are performed with each course of HDMTX. Close monitoring allows early intervention (e.g., increasing fluid hydration, discontinuing interacting drugs) and potentially reduces adverse effects. Using prior information about a patient’s MTX clearance to individualize a patient’s dosage of HDMTX is an added way to potentially reduce adverse effects when within-course dose adjustment is not feasible. However, we acknowledge that targeting doses based on clearance did not prevent all cases of severe nephrotoxicity. For those patients who do experience nephrotoxicity during or after a course of HDMTX, it is likely that intra-course clearance estimates with intra-course dose adjustments, as we previously described [13], would be more likely to prevent recurrence of severe nephrotoxicity than between-course adjustments.

Our current clinical approach to administering HDMTX in ALL is as follows. Because with the lower dose (~2.5 g/m^2^), the proportion of courses with delayed excretion was only modestly reduced (8.9 vs. 7.4 %) by targeting, we generally do not pharmacokinetically adjust doses of this lower dose of HDMTX unless the patient displays unstable or poor renal function. For patients receiving the equivalent of 5 g/m^2^, we adjust doses based on the pharmacokinetics of the prior course in most patients, because in the current study, we showed that individualizing doses reduced both the frequency of extremely high Cpss (1.3 vs. 7.3 %) and the frequency of delayed excretion (14.7 vs. 19.8 %). In the relatively rare patients with unstable clearance or renal function, we measure plasma concentrations early during the infusion as we described previously [13, 29] and adjust doses during the infusion to achieve the desired Cpss.

## Electronic supplementary material

Below is the link to the electronic supplementary material.

Supplementary material 1 (DOCX 504 kb)
