# Supplementary material for: Between-course targeting of methotrexate exposure using pharmacokinetically guided dosage adjustments
Source: Cancer Chemother Pharmacol. 2013 Jun 13;72(2):369–78. doi: 10.1007/s00280-013-2206-x (PMC3719000; doi:10.1007/s00280-013-2206-x)
Supplement: Supplementary file 1 — Supplementary material 1 (DOCX 504 kb) [file 280_2013_2206_MOESM1_ESM.docx]

**Supplemental material:**

**Between-course targeting of methotrexate exposure using pharmacokinetically-guided dosage adjustments**

Jennifer L. Pauley^1^, John C. Panetta^1,4^, Kristine R. Crews^1,4^, Deqing Pei^2^, Cheng Cheng^2^, John McCormick^1^, Scott C. Howard^3,4^, John Sandlund^3,4^, Sima Jeha^3,4^, Raul Ribeiro^3,4^, Jeffrey Rubnitz^3,4^, Ching-Hon Pui^3,4^, William E. Evans^1,4^, Mary V. Relling^1,4^

Departments of ^1^Pharmaceutical Sciences, ^2^Biostatistics, and ^3^Oncology, St. Jude Children's Research Hospital, Memphis, TN; ^4^Colleges of Medicine and Pharmacy, University of Tennessee, Memphis

Address reprint requests to Mary V. Relling, PharmD, Department of Pharmaceutical Sciences, St. Jude Children’s Research Hospital, 262 Danny Thomas Place, Memphis, TN 38105-3678; Phone: 901-595-2348; Fax: 901-525-8869; e-mail: mary.relling@stjude.org

**Supplemental Table 1: Leucovorin rescue dose schema**

**Window Therapy**

| Time from start of MTX Concentration | Plasma concentration thresholds for action | Recommended Leucovorin rescue |
| --- | --- | --- |
| 23 hr | if > 30 µM | For 24 hr infusion: check that hydration and alkalinization are adequate, check for nephrotoxic drugs, check creatinine  For 4 hr infusion: all of above plus consider glucarpidase and/or early LV rescue |
|  | > 50 µM | For either infusion schedule: All of above plus check another concentration, consider possible early LV rescue, obtaining glucarpidase, etc. |
| 42 hr | < 0.5 µM | protocol rescue (50 mg/m^2^ IV at hr 44, then 15 mg/m^2^ IV q6h x 7 doses) |
|  | 0.5 – 1 µM | 50 mg/m^2^ IV, then 30 mg/m^2^ IV q 6h x 7 doses |
|  | 1-2 µM | 60 mg/m^2^ IV, then 45 mg/m^2^ IV q 6 hrs |
|  | 2 - 5 µM | 60 mg/m^2^ IV q 6 hrs |
|  | 5-10 µM | 100 mg/m^2^ IV q 6 hrs |
|  | 10-20 µM | 200 mg/m^2^ IV q 6 hrs |
|  | >20 | Individualized |
| 66 hr | < 0.1 µM | per protocol and stop checking |
|  | 0.1-0.2 µM | 5 mg/m^2^ po/IV q 12 hrs and keep checking ~ q 24 hrs until MTX concentration < 0.1 |
|  | 0.2-0.5 µM | 15 mg/m^2^ po/IV q 6 hrs |
|  | 0.5-1.0 µM | 30 mg/m^2^ po/IV q 6 hrs |
|  | 1.0-2.0 µM | 45 mg/m^2^ q 6 hrs |
|  | 2-5 µM | 60 mg/m^2^ IV q 6 hrs |
|  | > 5 µM | Individualized |
| 92 hr and later | 0.1 - 0.2 µM | 5 mg/m^2^ q 12 hrs |
|  | 0.2 - 0.5 µM | 15 mg/m^2^ q 6 hrs |
|  | 0.5 - 1.0 µM | 30 mg/m^2^ q 12 hrs |
|  | > 1.0 µM | as above under 68 hrs |

**Consolidation Therapy**

| **Time from start of MTX Concentration** | **Plasma concentration thresholds for action** | **Recommended Leucovorin rescue** |
| --- | --- | --- |
| 23 hr--- **2.5 g/m^2^ (targeted to 33 µM)** | if > 45 µM | Check that hydration and alkalinization are adequate; check for nephrotoxic drugs; check creatinine, notify the house officer and floor nurse to be particularly vigilant with urine output. |
|  | > 150 µM | all of above plus obtain another blood sample for assay, consider possible early LV rescue, obtaining glucarpidase, etc. |
| 23 hr--- **5.0 g/m^2^ (targeted to 65 µM)** | if > 95 µM | Check that hydration and alkalinization are adequate; check for nephrotoxic drugs; check creatinine; notify the house officer and floor nurse to be particularly vigilant with urine output. |
|  | > 150 µM | all of above plus obtain another blood sample for assay, consider possible early LV rescue, obtaining glucarpidase, etc. |
| 42 hr | < 1 µM | protocol rescue (10 mg/m^2^ po q 6 hrs x 5 doses for LR and 15 mg/m^2^ IV/po x 5 doses for SR/HR) |
|  | 1-2 µM | 30 mg/m^2^ po/IV q 6 hrs |
|  | 2-5 µM | 50 mg/m^2^ IV q 6 hrs |
|  | 5-10 µM | 100 mg/m^2^ IV q 6 hrs |
|  | 10-20 µM | 200 mg/m^2^ IV q 6 hrs |
|  | >20 | Individualized |
| 66 hr | < 0.1 µM | per protocol and stop checking |
|  | 0.1-0.2 µM | 5 mg/m^2^ po/IV q 12 hrs and keep checking until MTX concentration < 0.1 |
|  | 0.2-0.5 µM | 15 mg/m^2^ po/IV q 12 hrs |
|  | 0.5-1.0 µM | 15 mg/m^2^ po/IV q 6 hrs |
|  | 1.0-2.0 µM | 30 mg/m^2^ po/IV q 6 hrs |
|  | 2-5 µM | 50 mg/m^2^ IV q 6 hrs |
|  | >5 µM | individualized |
| 92 hr and later | < 0.1 µM | stop LV and stop checking |
|  | 0.1-0.2 µM | 5 mg/m^2^ po/IV q 12 hrs |
|  | 0.2-0.5 µM | 15 mg/m^2^ po/IV q 12 hrs |
|  | 0.5 - 1.0 µM | 15 mg/m^2^ po/IV q 6 hrs |
|  | > 1.0 µM | as above under 68 hrs |

LV: Leucovorin. IV: intravenous. PO: oral. q 6 hrs: dose every 6 hours. q 12 hrs: dose every 12 hours.

**Supplemental Table 2: Patient Demographics**

|  |  | **Low Risk** | | **Standard/High Risk** | |
| --- | --- | --- | --- | --- | --- |
|  |  | **All patients** | **Only those who received Targeted MTX** | **All patients** | **Only those who received Targeted MTX** |
| **n** |  | 233 | 220 | 252 | 224 |
| **Sex** | **Male** | 114 | 109 | 160 | 137 |
|  | **Female** | 119 | 111 | 92 | 87 |
| **Self-Declared Race** | **Caucasian** | 191 | 180 | 197 | 175 |
|  | **African American** | 32 | 32 | 49 | 44 |
|  | **Other** | 10 | 8 | 6 | 5 |
| **Lineage/Ploidy** | **B lineage Hyperdiploid** | 102 | 95 | 18 | 16 |
|  | **B lineage Non-Hyperdiploid** | 131 | 125 | 160 | 143 |
|  | **T** | 0 | 0 | 74 | 65 |
| **Age (years)** | **Median (min, max)** | 4.0 (1.0, 18.5) | 3.9 (1.0, 18.5) | 8.3 (1.0, 18.9) | 8.3 (1.0, 18.9) |

**Supplemental Table 3: Univariate analysis of clinical features vs grade 3 or greater gastrointestinal toxicity (A) in patients on the low risk arm and (B) patients on the standard/high risk arm.**

**(A)**

|  | | **Parameter Estimates** | | **Odd Ratio** | |  |
| --- | --- | --- | --- | --- | --- | --- |
| **Clinical Variables** | **Clinical Level** | **Estimate** | **Std err** | **Odd Ratio** | **95% CI of OR** | **P Values** |
| MTX Cpss | MTX Cpss | 0.0464 | 0.0201 | 1.05 | 1.01 to 1.09 | 0.0210 |
| MTX 42 hr concentration | MTX 42 hr concentration | 0.1701 | 0.1515 | 1.19 | 0.88 to 1.60 | 0.2614 |
| Log-Total Leucovorin dose | Log-Total Leucovorin dose | 0.3758 | 0.7826 | 1.46 | 0.31 to 6.75 | 0.6311 |
| Targeting success | In Target | -0.2593 | 0.8149 | 0.77 | 0.16 to 3.81 | 0.7504 |
|  | Over Target | 1.0121 | 0.8203 | 2.75 | 0.55 to 13.73 | 0.2173 |
|  | Under Target |  |  |  |  |  |
| MTX Delayed Excretion | MTX delayed | 0.4893 | 0.7666 | 1.63 | 0.36 to 7.33 | 0.5233 |
|  | Other |  |  |  |  |  |

**(B)**

|  | | **Parameter Estimates** | | **Odd Ratio** | |  |
| --- | --- | --- | --- | --- | --- | --- |
| **Clinical Variables** | **Clinical Level** | **Estimate** | **Std err** | **Odd Ratio** | **95% CI of OR** | **P Values** |
| MTX Cpss | MTX Cpss | 0.0083 | 0.0072 | 1.01 | 0.99 to 1.02 | 0.2491 |
| MTX 42 hr concentration | MTX 42 hr concentration | 0.0751 | 0.0452 | 1.08 | 0.99 to 1.18 | 0.0970 |
| Log-Total Leucovorin dose | Log-Total Leucovorin dose | 1.0741 | 0.2237 | 2.93 | 1.89 to 4.54 | <.0001 |
| Targeting success | In Target | 0.1378 | 0.4537 | 1.15 | 0.47 to 2.79 | 0.7614 |
|  | Over Target | 0.2439 | 0.4926 | 1.28 | 0.49 to 3.35 | 0.6206 |
|  | Under Target |  |  |  |  |  |
| MTX Delayed Excretion | MTX delayed | 1.1068 | 0.3759 | 3.02 | 1.45 to 6.32 | 0.0032 |
|  | Other |  |  |  |  |  |

MTX: methotrexate. Cpss: Steady-State methotrexate concentration. In Target: achieved Cpss within 20% of targeted Cpss. Over Target: achieved Cpss greater than 20% above targeted Cpss. Under Target: achieved Cpss below 20% of targeted Cpss. MTX delayed: methotrexate concentration greater than 1 µM at 42 hours. Std err: Standard Deviation. CI: Confidence Interval. OR: Odds Ratio. The Odds Ratio and corresponding p-value were determined using a univariate generalized estimating equation model. MTX Cpss, MTX 42 hr concentration, and log-total leucovorin dose were analyzed as continuous variable data; targeting success and MTX delayed excretion were analyzed as categorical variables.

**Supplemental Figure 1: Methotrexate Dose Individualization Scheme at Consolidation**

Low Risk Arm

Estimate MTX CL from previous HDMTX course

Use predicted MTX CL to predict MTX dose needed to achieve a Cpss of 33 µM

Use estimated dose

Maximum increase or decrease of 50% of previous dose

Estimated dose **is** within 50% of previous dose.

Estimated dose is **NOT** within 50% of previous dose.

Standard/High Risk Arm

Estimate MTX CL from previous HDMTX course

Use predicted MTX CL to predict MTX dose needed to achieve a Cpss of 65 µM

Use estimated dose

Maximum increase or decrease of 50% of previous dose

Estimated dose **is** within 50% of previous dose.

Estimated dose is **NOT** within 50% of previous dose.

Previous MTX CL ≤ 125 ml/min/m^2^

Previous MTX CL > 125 ml/min/m^2^ or first 55 patients.

Use previous MTX CL, CRE, BILI, and SGPT to predict MTX CL for current course.^*^

Use previous MTX CL to predict MTX CL for current course.

Use previous MTX CL to predict MTX CL for current course.

MTX: methotrexate. CL: clearance. HDMTX: High Dose methotrexate. CRE: creatinine. BILI: bilirubin. SGPT: Serum Glutamic Pyruvic Transaminase. Cpss: Steady-State methotrexate concentration.

* Log(MTX CL) = 4.349 + 0.1152·Log(Previous MTX CL) - 0.3422·CRE – 0.2390·BILI – 0.000582·SGPT

**Supplemental Figure 2: Methotrexate clearance (MTX CL) vs serum chemistries obtained within 24 hours before targeted courses during consolidation in standard/high risk patients (n=683 courses in 187 patients). Circles are the individual measurements and the solid line is the best fit line. (A) creatinine (r^2^=0.037, p<10^-4^), (B) bilirubin (r^2^=0.048, p<10^-4^) and (C) SGPT (Serum Glutamic Pyruvic Transaminase) (r^2^=0.014, p<2.5Χ10^-3^).**

**(A)**


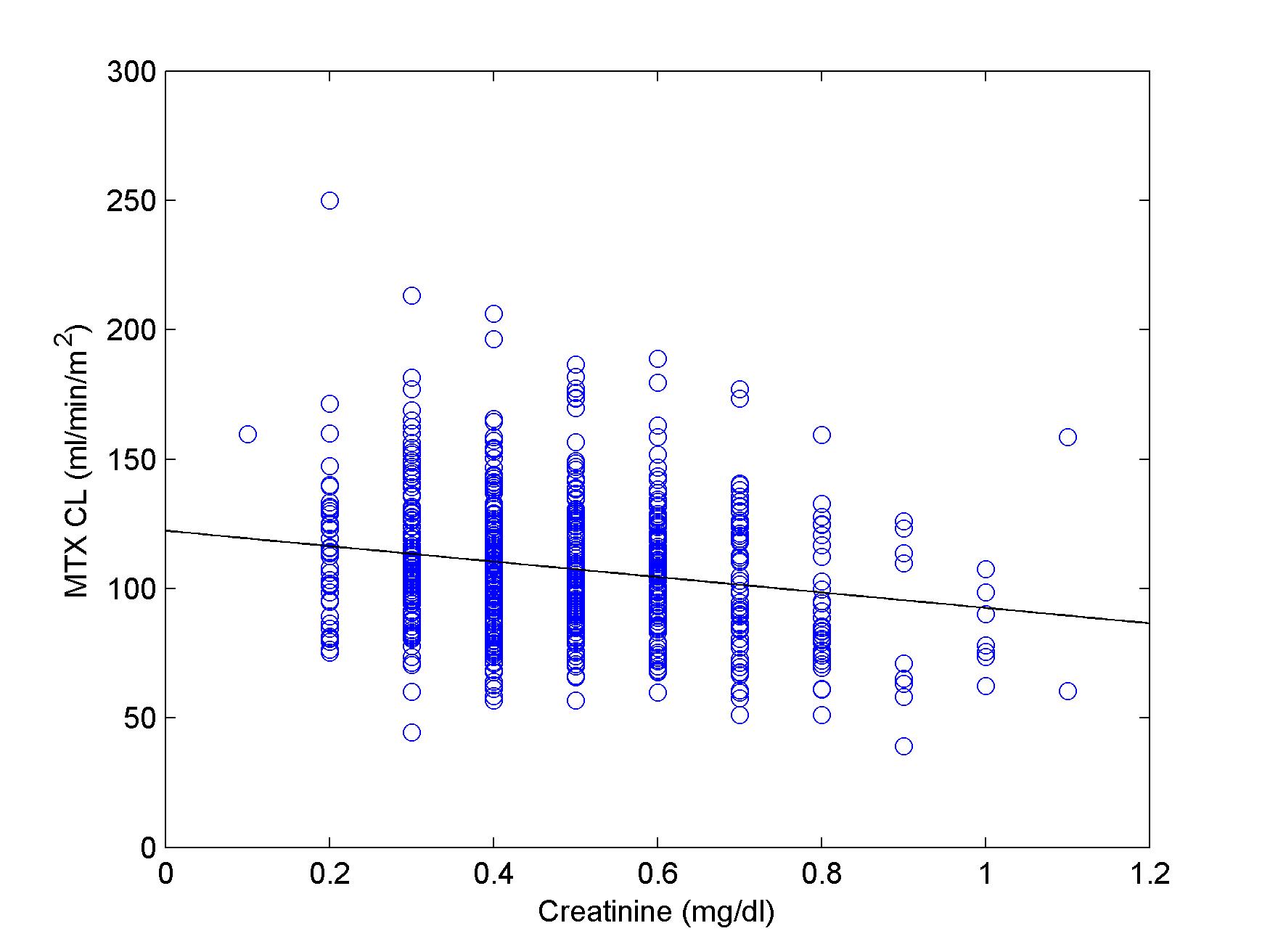


**(B)**

**
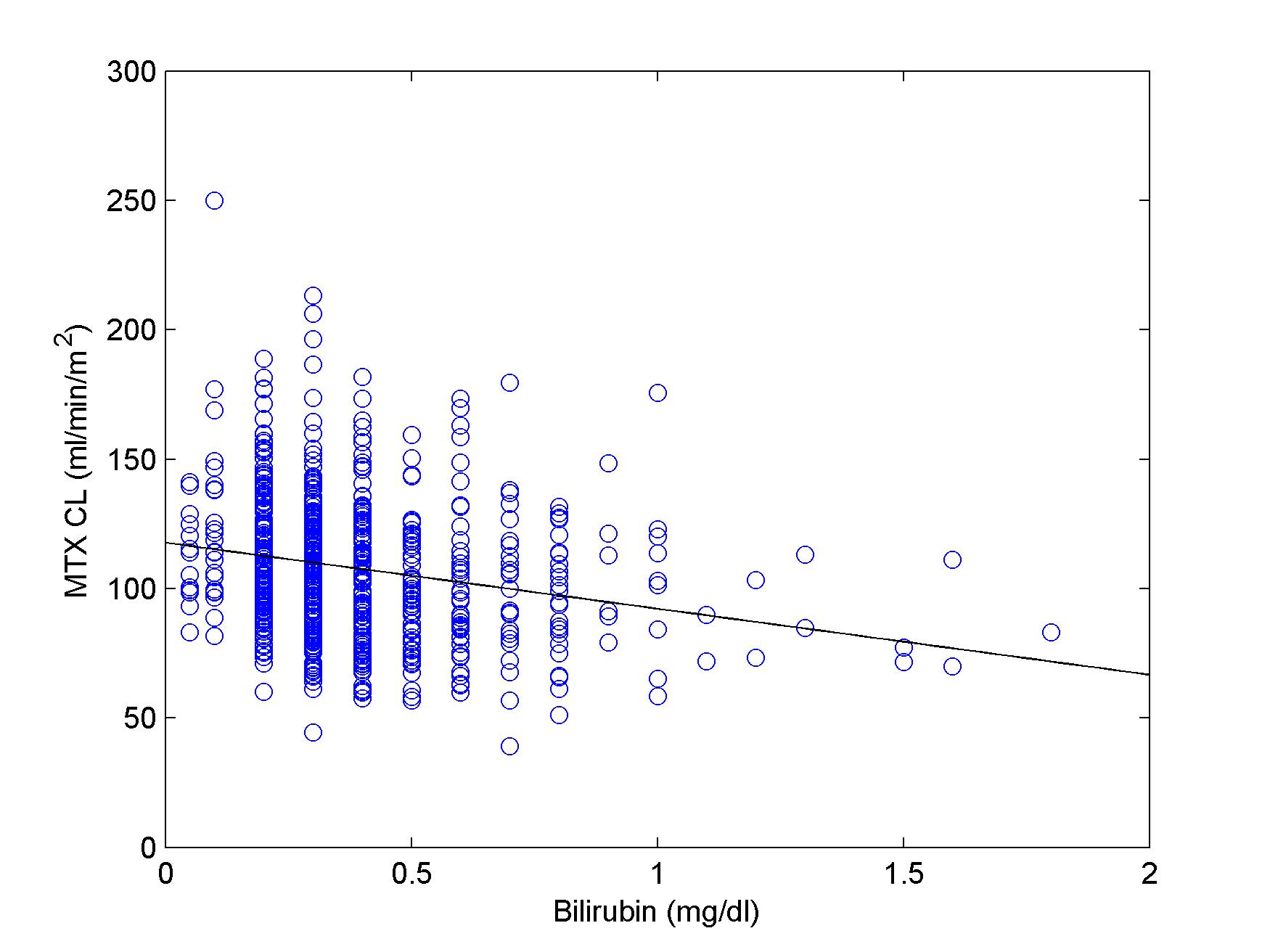
**

**(C)**

**
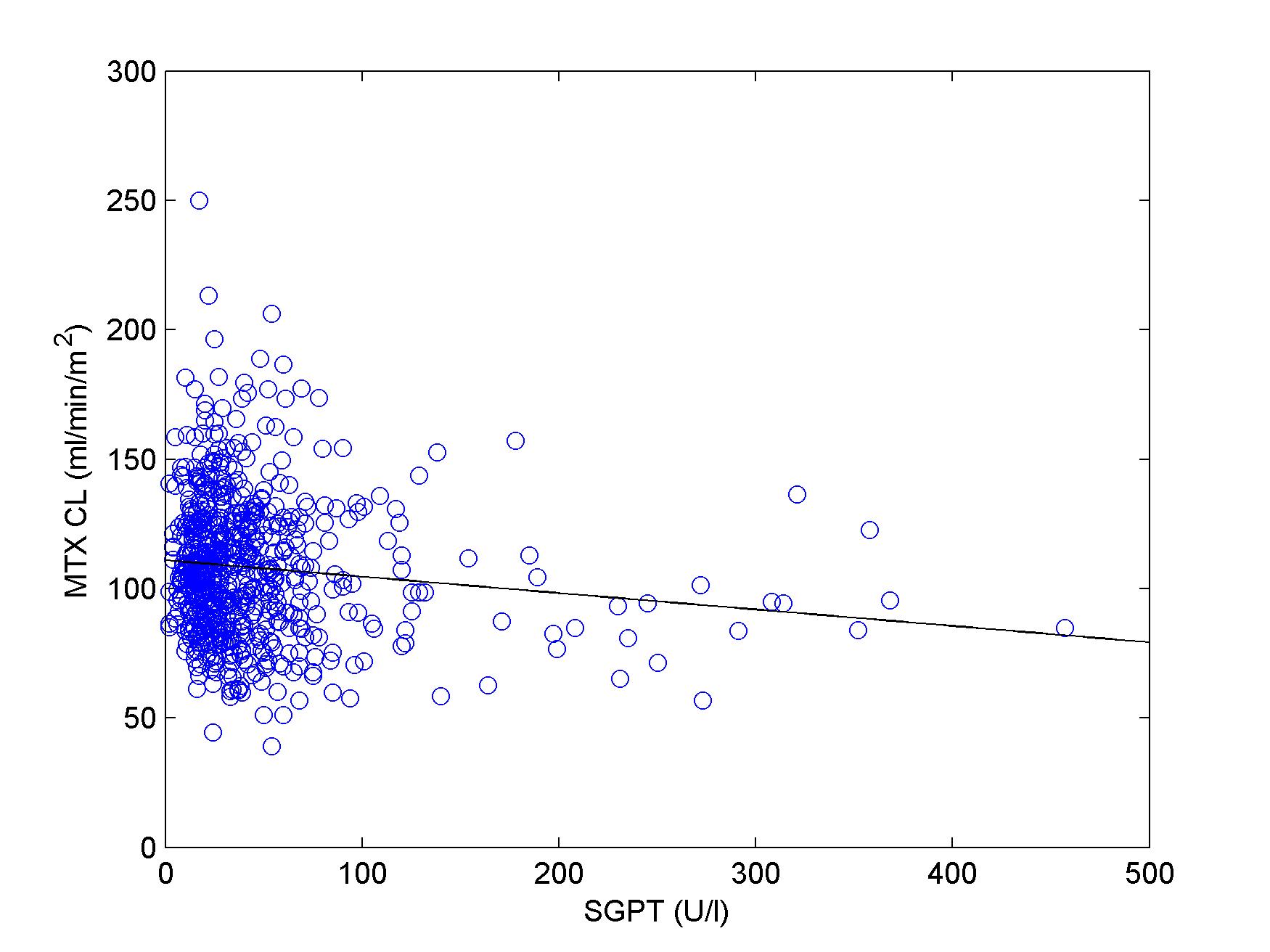
**
